# Is DNA Damage Response Ready for Action Anywhere?

**DOI:** 10.3390/ijms130911569

**Published:** 2012-09-14

**Authors:** Mariona Terradas, Marta Martín, Laia Hernández, Laura Tusell, Anna Genescà

**Affiliations:** Department of Cell Biology, Physiology and Immunology, Universitat Autònoma de Barcelona, 08193 Bellaterra (Cerdanyola del Vallès), Spain; E-Mails: marta.martin@uab.cat (M.M.); laia.hernandez@uab.cat (L.H.); laura.tusell@uab.cat (L.T.); anna.genesca@uab.cat (A.G.)

**Keywords:** micronuclei, DSB repair, NER pathway, chromosome instability

## Abstract

Organisms are continuously exposed to DNA damaging agents, consequently, cells have developed an intricate system known as the DNA damage response (DDR) in order to detect and repair DNA lesions. This response has to be rapid and accurate in order to keep genome integrity. It has been observed that the condensation state of chromatin hinders a proper DDR. However, the condensation state of chromatin is not the only barrier to DDR. In this review, we have collected data regarding the presence of DDR factors on micronuclear DNA lesions that indicate that micronuclei are almost incapable of generating an effective DDR because of defects in their nuclear envelope. Finally, considering the recent observations about the reincorporation of micronuclei to the main bulk of chromosomes, we suggest that, under certain circumstances, micronuclei carrying DNA damage might be a source of chromosome instability.

## 1. Introduction

To answer to the diversity of DNA lesions, cells have developed a complex defense system in order to detect them, signal their presence and promote their repair. This system, known as the DNA damage response (DDR), is a hierarchical cascade of proteins composed of sensors, mediators, transducers and effectors. The front line of the DDR system is made of the sensors that recognize the damage and activate the transducers. These transducers are in charge of amplifying the damage signal through the activation of mediators. Similarly, transducers activate the effectors that coordinate the temporary delay of cell cycle progression, which is needed for DNA damage repair, and, sometimes, the activation of apoptosis or senescence. In light of this complex scenario, cells must overcome important spatio-temporal limitations to swiftly sense the DNA lesions and initiate the correct signaling and repair programs to restore genome integrity. However, there is evidence that indicate that DDR is impaired when lesions are located inside micronuclei. In this text, we have reviewed the current up-to-date research on the presence of DDR factors inside micronuclei. We have focused on the works that supported the conclusion that micronuclei are almost incapable of generating an effective DNA damage response. Specifically, we review different studies that analyze the presence of factors involved in the response to DNA double strand breaks (DSBs) and of those involved in the repair of helix-distorting base lesions in micronuclei.

## 2. Micronuclei

Micronuclei or Howell-Jolly bodies are structures morphologically identical to the cell nucleus but smaller in size. These structures were first observed by haematologists William Howell (1890) and Justin Jolly (1905). Originally, its presence was explained by vitamin deficiency and it was not until the 1940s that micronuclei were related to mutagen exposition and cancer [1]. Nowadays, it is well known that micronuclei mainly originate from the loss of acentric fragments, the loss of whole chromosomes during mitosis and, sometimes, from the elimination of extrachromosomal double minutes (DMs). Accordingly, micronuclei are considered genetic markers of cancer susceptibility.

When cells are exposed to clastogenic agents, chromosome breaks are formed. In this situation, if the DNA damage is massive, some DSBs might be misrepaired or left unrepaired, resulting in the formation of acentric fragments. These fragments do not efficiently integrate in any of the daughter nuclei because they are incapable of attaching to the spindle fibers and are left behind during mitosis. During telophase, they are enclosed by the nuclear envelope and arise as micronuclei in the next cell cycle. If two broken chromosome ends are mis-repaired and fuse with each other, a dicentric chromosome can be formed. Dicentric chromosomes can also be formed by the fusion of two unprotected ends resulting from telomeric dysfunction [2,3], or by the fusion of an unprotected end and a broken chromosome [4]. The two centromeres of the dicentric chromatids can be pulled to opposite poles during the next anaphase, forming a chromosomal bridge that is frequently resolved by breakage. This breakage may result in the formation of acentric fragments that, at the end of mitosis, arise as micronuclei [5] or as nuclear blebs, which are micronucleus-like bodies physically connected to the nucleus by a chromatinic filament [6,7]. The broken bridge often constitutes a source of chromosomal instability, as the resulting unprotected chromosomal ends are susceptible to suffering further reorganization. The new chromosome reorganization provides the basis of another breakage-fusion-bridge (BFB) type of mitotic disturbance. The BFB cycle was first described in 1941 by Barbara McClintock [8], and is nowadays considered one of the main causes of chromosomal instability in cancer cells.

Alternatively, micronuclei can contain a whole chromosome arising from anaphase loss. This loss is a consequence of defects during the mitotic spindle assembly, misregulation of the spindle assembly checkpoint or the presence of supernumerary centrosomes [1]. It has also been shown that micronuclei can contain whole chromatids derived from the merotelic attachment of chromosomes to the mitotic spindle. In this sense, when a single kinetochore is connected to microtubule bundles coming from both poles, the affected chromatid lags behind from the bulk of chromosomes and, at the end of mitosis, is enclosed into a micronucleus [9]. Therefore, micronuclei with whole chromosomes can be induced by using drugs that interfere with the proper assembly of the mitotic spindle, *i.e.*, they appear after cell treatment with aneugenic agents. Moreover, it has recently been suggested that proliferation-dependent telomere dysfunction could be an underlying condition for erroneous chromosome segregation into micronuclei [6,7]. According to the authors, the mechanical tension of bridged end-to-end fused chromosomes can result in abnormal segregation patterns and micronuclei formation. This may occur when the distorted dicentric chromatid resists as an unbroken unit, eventually detaching from both spindle poles.

As outlined before, micronuclei can also arise from the elimination of DMs, which are autonomously replicating acentric chromatin bodies composed of circular DNA that do not require telomeric ends. The presence of DMs has important implications for the cancer cell phenotype as DMs appear in various types of human cancer cells and are never observed in normal cells [10]. DM-type micronuclei formation can take place both in mitosis and interphase. Time-lapse experiments with cell lines expressing green fluorescent protein (GFP)-tagged DMs’ chromatin have allowed the observation that, during mitosis, DMs aggregate to be later left behind and are finally sequestered into micronuclei [11]. Alternatively, DMs could be extruded from the main nucleus by means of a nuclear bleb also during mitosis. This phenomenon has been observed after cell treatment with hydroxyurea that, at low concentrations, induces DNA breaks instead of inhibiting DNA replication [12].

According to the different origin of micronuclei, their presence in cells is not only an indicator of mutagenic agent exposures but also an indicator of ongoing chromosome instability. This type of genomic instability is defined as a dynamic state capable of generating a high rate of alterations in chromosomes as, for example, changes in chromosome number and alterations in chromosome structure [13]. Considering that acquisition of genomic instability is an early step in carcinogenesis [14], it is not surprising that increased frequencies of micronuclei have been found in patients with cancer or with pre-neoplasic lesions [15]. Most importantly, Bonassi *et al.* provided evidence that high micronucleus frequencies in peripheral blood lymphocytes of healthy individuals are predictive of higher cancer risks, suggesting that increased micronuclei formation is associated with early events in carcinogenesis [16,17].

## 3. Response to Double Strand Breaks in Micronuclear DNA

When a DSB is induced, the protein complex composed of MRE11, RAD50 and NBS1 (MRN complex) detects the lesion and binds to the DNA through MRE11. It also acts as a bridge between the two broken DNA ends, keeping them in close proximity to facilitate a proper repair [18]. Subsequently, latent ATM dimers are activated and bind the MRN complex through the *C*-terminal part of NBS1 [19]. The active monomers of ATM transduce the signal by means of the phosphorylation of different targets, including those factors responsible of the cell cycle regulation, as well as others that ultimately repair the DNA damage. H2AX, a histone H2A variant that comprises 10%–15% of total cellular H2A in higher eukaryotes, is the first substrate of ATM and once phosphorylated on S139 becomes γH2AX [20,21]. The key function of γH2AX is to provide a high-affinity binding site for the MDC1 protein, which is then recognized by the MRN complex, recruiting more ATM molecules to the site and thus extending the H2AX phosphorylation to 1–2 megabases around the lesion [22]. The recruitment kinetics of these factors to foci following exposure to DSB-inducing agents is fast, reaching maximal accumulation within a few minutes. After 1–2 min, this is followed by a second wave of protein accumulation at sites of DNA damage that includes 53BP1 and BRCA1 [23]. Their retention at the DNA lesion depends on the action of RNF8, an ubiquitin ligase that initiates an intricate and tightly regulated ubiquitylation cascade of histones H2A and H2AX at the DSB-flanking chromatin [24]. The accumulation of all these proteins at the damaged DNA site leads to the formation of ionizing radiation-induced foci (IRIF). IRIF maintenance until repair is critical for the maintenance of genomic stability, because, as it has been explained, they control various DNA and chromatin transactions and stimulate DNA repair. There are two main pathways in charge of DSB repair: the non-homologous end joining (NHEJ) and the homologous recombination (HR) [25]. HR is well known for its fidelity as it uses the non-damaged sister chromatid as a template and thus is only active during the S and G2 phases. On the other hand, NHEJ is active through all the cell cycle and for this reason it is considered the main DSB repair pathway in humans [26]. NHEJ consists on the processing and re-joining of DNA ends with few or none end-to-end complementarity and thus is considered more error prone than HR [27].

Our laboratory investigated the presence of DSB repair markers in the micronuclear DNA in primary human fibroblasts exposed to γ-rays. Specifically, by means of γH2AX foci detection and colocalization with 53BP1 and MRE11, we observed that only some micronuclear DSBs recruited these DDR factors [28]. This observation suggests that the micronuclear environment is hostile for DSB repair. Furthermore, the initial approach might have underestimated the micronuclear DDR defect. This is due to the possibility that some repair factors observed inside micronuclei were recruited to damaged chromatin previous to the micronucleus formation, instead of being imported through the micronuclear envelope after DSB formation. Since ionizing radiation is a clastogenic agent, radiation-induced micronuclei mainly derive from acentric fragments and from anaphase bridge breakage, which could potentially trigger recruitment of repair factors to DSB lesions before the formation of the micronucleus. However, observation of 53BP1 in micronuclei reflects a real recruitment to the lesion after micronuclear envelope formation, as 53BP1 does not associate to DSBs in mitotic chromosomes [29,30]. Similarly to our findings, Yoshikawa *et al.* found low colocalization of 53BP1 and γH2AX in radiation-induced micronuclei of normal and tumorigenic cells [31]. Very recently, Crasta *et al.* also observed a very poor recruitment of 53BP1 in micronuclear DSBs induced after treatment with the replication inhibitor aphidilcolin [32]. Moreover, Medvedeva *et al.* claimed the absence of 53BP1, MRE11, RAD50 and RAD17 in the micronuclear γH2AX foci [33]. On the other hand, several studies reported the presence of phosphoinositade 3 (PPI3)-kinases in micronuclear DSBs. Medvedeva and Yoshikawa found that ATM and DNA-PKcs colocalized with γH2AX [31,33]. ATR, which is another member of the PPI3-kinases family, was found in the micronuclear DSBs induced by aphidilcolin [32]. Surprisingly, the repair factors supposed to be recruited to the lesions after activation of the kinases were frequently absent in the micronuclear lesions. All these observations lead to the conclusion that DNA damage response factors are inconsistently detected in micronuclear DSBs (Table 1). While there is a low or nonexistent recruitment for most of the repair factors, some core kinases seem to be loaded more efficiently but, once at the site of lesion, they do not seem to trigger the proper recruitment of downstream factors in their pathway. We conclude, consequently, that DSBs sequestered in micronuclei induce a defective-DNA damage response.

While trying to assess whether newly formed whole-chromosome containing micronuclei spontaneously develop DNA damage, Crasta *et al.* observed that neither the main nucleus nor the newly formed micronuclei showed significant DNA damage (measured by H2AX phosphorylation) during the subsequent G1 phase [32]. After the following S-phase, H2AX phosphorylation was detected in both the micronuclei and the main nucleus. This phosphorylation is consistent with typically higher levels of γH2AX during S-phase [34]. Although the nuclear H2AX phosphorylation is completely removed in nuclei at the G2-phase, micronuclei still show G2-phase γH2AX labelling. Therefore, the authors suggested that the micronuclear DNA damage might be a consequence of DNA replication. By blocking DNA replication, they observed that acquisition of DNA damage in micronuclei was abolished and thus, demonstrated their hypothesis. Moreover, they also observed that, while the first step of DNA replication is equally efficient in the micronuclei as in the main nucleus, micronuclei fail to complete a DNA replication round. Therefore, Crasta *et al.* demonstrated that aberrant DNA replication is a source of DNA damage in the micronuclear environment [32]. The authors also reported that micronuclei had clear signal for γH2AX and ATR, but downstream constituents of the DNA damage response such as 53BP1 were not efficiently recruited. As micronuclei seem to be unable to process DSBs, the replication-dependent DNA damage will remain unrepaired in the micronucleus and new breaks may accumulate with every DNA replication cycle.

Micronuclei are not the only site in the cell where repair of DSBs is impaired as there are regions of the nucleus where chromatin organization hampers a rapid response to DSBs. In this sense, in 2007, Kim *et al.* found that heterochromatin is refractory to γH2AX modification in yeast and mammals, *i.e.*, H2AX is not efficiently modified in response to DSB [35]. Karagiannis *et al.* observed by ChIP analysis that chromatin containing satellite 2 and α-satellite, which are constituents of centromeric heterochromatin, are resistant to formation of phospho-H2AX foci by ionizing radiation [36]. Moreover, Cowell *et al.* showed that local phosphorylation of H2AX occurs preferentially in euchromatic regions of the genome following X-ray, according to immunofluorescence microscopy.

Importantly, H2AX was efficiently phosphorylated in heterochromatin during DNA replication, when heterochromatin organization is more relaxed [37]. Thus differences between euchromatin and heterochromatin in γH2AX foci induction by X-rays might be related to differences in condensation state rather than heterochromatin being refractory to DSB induction. Concerning this issue, different studies showed how chromatin configuration does not affect radiosensitivity and thus DSBs are linearly induced in heterochromatin [38,39]. From the analysis of IRIF persisting in the absence of ATM, it was observed that IRIF were located at the close periphery of heterochromatin domains, but were rarely located within these domains [40]. Goodarzi *et al.* reviewed these and other results and concluded that higher order heterochromatin superstructure restricts, although may not completely block, the signal expansion at DSBs. As a result of the DSBs’ expanding signal, heterochromatin slightly relaxes and promotes its movement to the periphery, where signal expansion proceeds [41]. Therefore, similarly to the differences between the main nucleus and the micronucleus, DSB repair in heterochromatin occurs with slower kinetics and is less effective than in euchromatin [42].

## 4. NER Factors in Micronuclei

**Figure 1:**
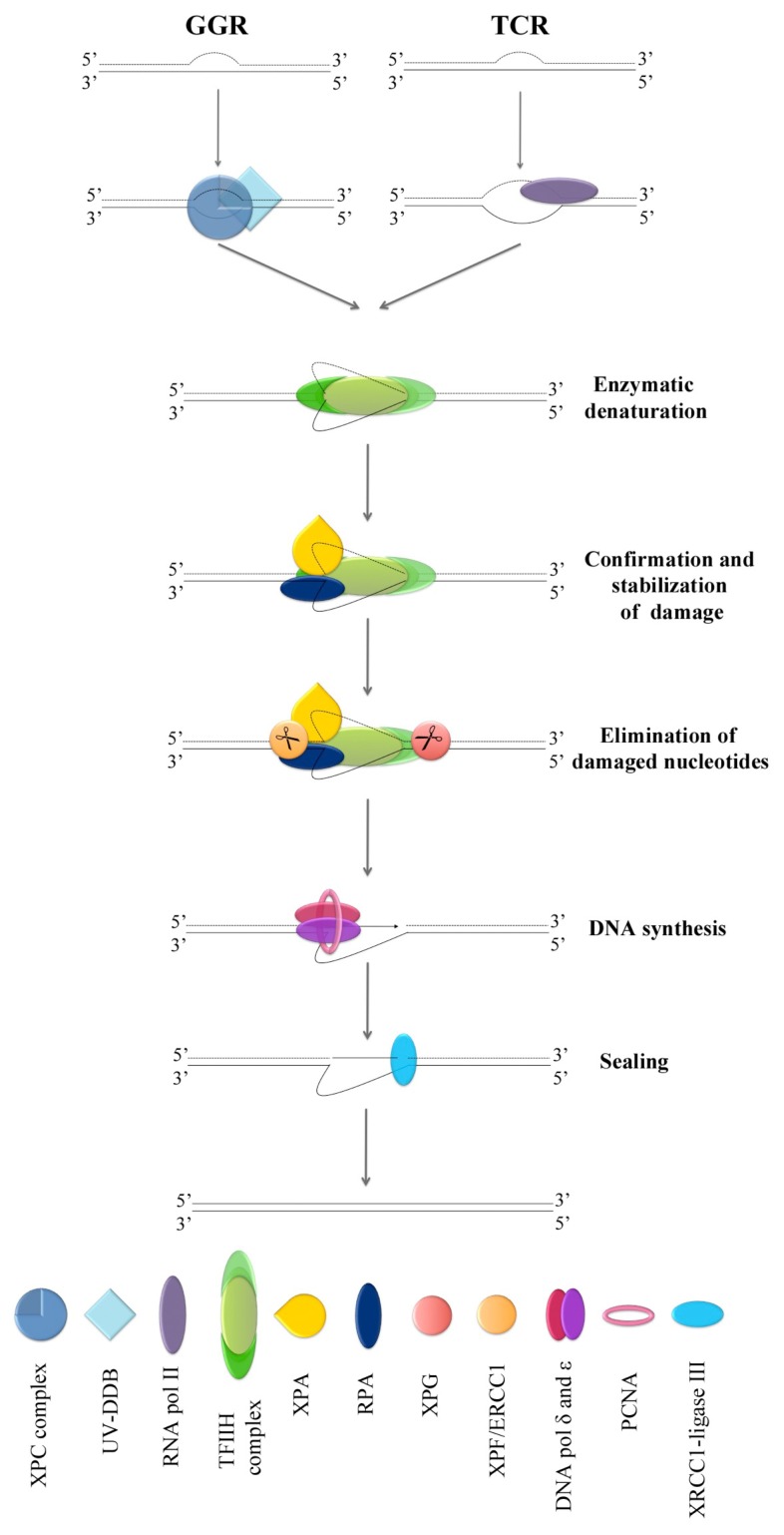
Model for nucleotide excision repair pathway including both subpathways: the global genomic repair (GGR) (left) and the transcription-coupled repair (TCR) (right).

Although highly deleterious, DSBs are not the only lesions that DNA may suffer. Helix-distorting base lesions are indeed very frequent and are repaired by the nucleotide excision repair (NER) pathway, whose versatility accounts for the repair of a wide variety of lesions. In this sense, NER is in charge of repairing the DNA damage formed upon exposure to the UV radiation from sunlight and numerous bulky DNA adducts induced by mutagenic chemicals from the environment or by cytotoxic drugs used in chemotherapy [43]. The NER pathway consists of several steps, but the presence of distortion in the structure of the double helix or the chemical modification of the DNA is indispensable for its activation. NER can be subdivided into two different subpathways depending on the transcription state of the damaged genes: the global genomic repair (GGR) and the transcription-coupled repair (TCR) (Figure 1). Whereas in the first one the damage is detected by the collective action of the protein UV-DDB and the XPC containing complex, in the second one the lesions are recognized by the RNA polymerase II itself [44]. Once the lesions have been detected, both subpathways converge and the later steps of repair are conducted by a common set of protein factors [45,46]. At this point, the multi-functional complex TFIIH is recruited to the DNA damage site and unwinds the DNA to allow the other factors to reach the lesion. XPB and XPD are the helicase subunits of the TFIIH complex responsible for the 30 bp opening of the DNA, which is immediately stabilized by RPA through its binding to the undamaged strand [47,48]. Simultaneously, XPA binds to the 5′ end of the lesion, where it confirms the presence of damage by probing for abnormal backbone structure. When damage is absent, XPA aborts NER [45]. The stabilization of the damaged DNA allows the proper orientation of the two endonucleases that remove the damaged nucleotides: XPG and the complex XPF/ERCC1. While XPG cuts at the 3′ end of the lesion, XPF/ERCC1 cuts at the 5′ end, leaving a 25–30 nucleotides gap [49]. The gap is then filled by the DNA polymerases δ and ɛ, joined together by the sliding clamp PCNA, and sealed by the XRCC1-ligase III [50].

In our laboratory we aimed to analyze whether factors from the NER pathway could be recruited inside micronuclei. To achieve this goal, γ-radiation-induced micronuclei were exposed to UV light to induce, as stated before, helix-distorting base lesions, which are processed by the NER pathway [51]. To assure that members of the two NER subpathways were included in the analysis, we studied the presence of XPC and XPA. While XPC detects the damage in the GGR, XPA participates in both subpathways stabilizing the lesion. In contrast to the cell nucleus, where these proteins accumulated properly to the nuclear photolesions, we observed that less than a half of the micronuclei showed XPC after UV-irradiation, only one in four exhibited XPA and less than 20% of UV-irradiated micronuclei showed both NER factors. Therefore, compared to the nuclear capacity to recruit NER factors, XPA and XPC are significantly less capable of reaching the photolesions when they are located at the micronuclear chromatin. In order to obtain a more dynamic picture of the micronuclear NER defect, we analyzed NER factor changes through time after UV exposure. While the number of XPC positive micronuclei increased over time after UV-exposure, the number of XPA positive micronuclei remained constant. These results reflect the dynamics of these proteins inside the cell. XPA resides in the nucleus and does not shuttle between cellular compartments. In order to accumulate at photolesions XPA has to be in the nucleus before UV-irradiation [52]. As a consequence, its import to the nucleus takes place after nuclear envelope reorganization at the end of mitosis with the bulk of the nuclear proteins. This would explain why the number of XPA positive micronuclei remains constant over time after UV-irradiation. Under unchallenged conditions, XPC freely shuttles between the cytoplasm and the nucleus until exposure to UV-C. After cell damage, the export of XPC is impeded leading to a temporary accumulation of XPC in the nucleus [53]. This would explain why the number of XPC positive micronuclei increase over time. On the whole it was demonstrated that UV-induced lesions in the micronuclear DNA are less capable of triggering an adequate DDR than nuclear injuries are.

## 5. Nuclear Envelope Defects Hamper the Recruitment of DDR Factors to Micronuclear DNA Lesions

As outlined before, even if our results indicate that some DDR factors are capable to get into micronuclei after their formation, the low frequency of 53BP1 and γH2AX colocalization and the low number of XPC positive micronuclei suggests the presence of a general impediment to the import of repair factors into the micronucleus. In this sense, the high colocalization of γH2AX and 53BP1 in nuclear blebs reinforces this hypothesis: whereas the 62% of γH2AX foci colocalize with 53BP1 in the nuclear blebs, only the 14% of γH2AX foci showed 53BP1 in micronuclei [28].

If there is an impediment to import proteins to the micronucleus, it should be found in the micronuclear envelope. Considering that the micronuclear membrane is structured in the same way as the nuclear membrane, it should be composed of two lipid bilayers fused to form the nuclear pore complexes (NPC), which consist of multiple copies of different nucleoporines that mediate macromolecule traffic through the envelope. Underneath the membrane, there is the nuclear lamina: a web of intermediate filaments (lamins A, B1, B2 and C) that stabilizes the nucleus and helps to organize the chromatin.

**Figure 2:**
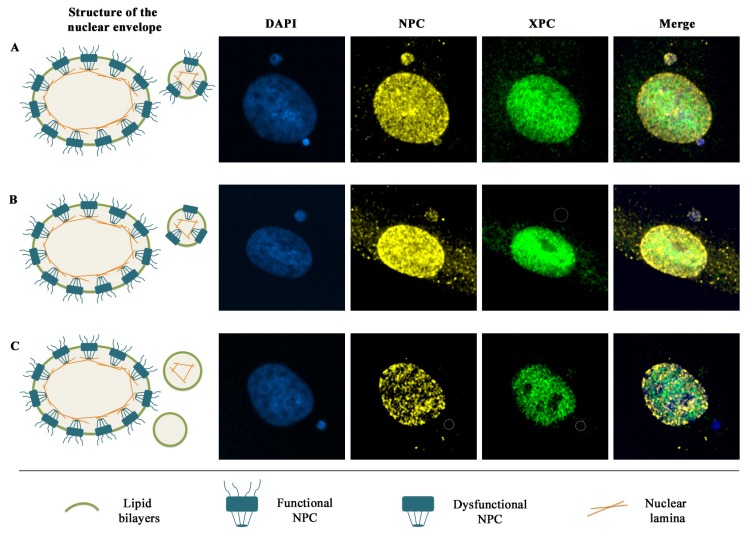
Model relating the structure and functionality of the nuclear envelope to the import of XPC (*Xeroderma pigmentosum* group C) into the micronucleus: Diagram of the structure of the micronuclear envelope (left); Double nuclear pore complexes (NPC)/XPC immunofluorescence carried out in our laboratory (right). (**A**) Micronucleus with a proper envelope labeled with both NPC and XPC. (**B**) Micronucleus with dysfunctional NPCs labeled with NPC but it does not show XPC labeling. (**C**) Micronucleus lacking NPCs and, in some cases, without lamina as it does not display either NPC nor XPC.

To determine whether the low presence of DDR factors in micronuclei is a consequence of defects in the micronuclear envelope, the presence of nucleoporines (rich in FXFG repeats) and lamin B1 in radiation-induced micronuclei was analyzed by immunofluorescence assay [54]. While the main part of micronuclei showed lamin B1, only 30% of them exhibited nucleoporines. This result pointed to the absence of nucleoporines as the main cause of import defects, which was finally demonstrated when the presence of XPC and nucleoporines was analyzed in the same experiment and no XPC positive micronucleus was devoid of nucleoporines. In any case, some micronuclei showing nucleoporines were XPC negative, suggesting that the NPCs detected at these micronuclei were dysfunctional (Figure 2).

Micronuclei are much smaller and contain less DNA than the main nucleus. Our hypothesis is that these characteristics hinder the formation of the micronuclear membrane and would contribute to the low number of micronuclei with a proper envelope. To back up the previous hypothesis up we established three categories of micronuclear size: small, medium and large. We also classified micronuclei depending on the presence or absence of XPC and Nups. The three different categories of micronuclei showed the three possible patterns of XPC and Nups labeling: micronuclei positive for both XPC and Nups, micronuclei only showing Nups, and micronuclei devoid of both XPC and Nups. However, the frequency of Nups positive micronuclei was higher in the large micronucleus category, while the frequency of Nups negative micronuclei was higher in the small and medium ones. Therefore, the presence of Nups seems to depend on the size of micronuclei and, by logical extension, on the amount of DNA contained in the micronucleus. This is not surprising if we consider that post-mitotic nuclear pore assembly is initiated by chromatin binding of several nucleoporins to easily accessible DNA sequences [55]. The probability of finding these DNA sequences where nucleoporins can bind decreases in the micronuclei with less DNA content, and this could be responsible for the absence or incomplete formation of the nuclear pores in their micronuclear envelope.

The micronuclear envelope defect observed in our laboratory is not unique. By means of electron microscopy, morphological observations of both micronuclei induced with aneugenic treatments and micronuclei found in cancer cells showed that the envelope of micronuclei is abnormal or incomplete compared to that of nuclei [5,56,57]. More recently, two different laboratories demonstrated that the micronuclear import capacity is strongly reduced in micronuclei derived from nocodazole treatment [32] and micronuclei derived from anaphase-bridge breakage [5]. To address this issue, Crasta *et al.* increased calcium level in the cytoplasm to induce translocation of NFATc1 transcription factor to both nucleus and micronucleus. Results showed that micronuclei were partially defective for the import of this transcription factor. Instead, Hoffelder *et al.* measured the nuclear uptake of exogenous glucocorticoid receptor after addition of dexamethasone, demonstrating a markedly diminished receptor uptake for micronuclei compared to nuclei. As a conclusion, all these results demonstrate that deficient DDR in micronuclei is caused by nuclear envelope defects.

## 6. Conclusions: Could Be Micronuclei a Source of Chromosome Instability?

**Figure 3:**
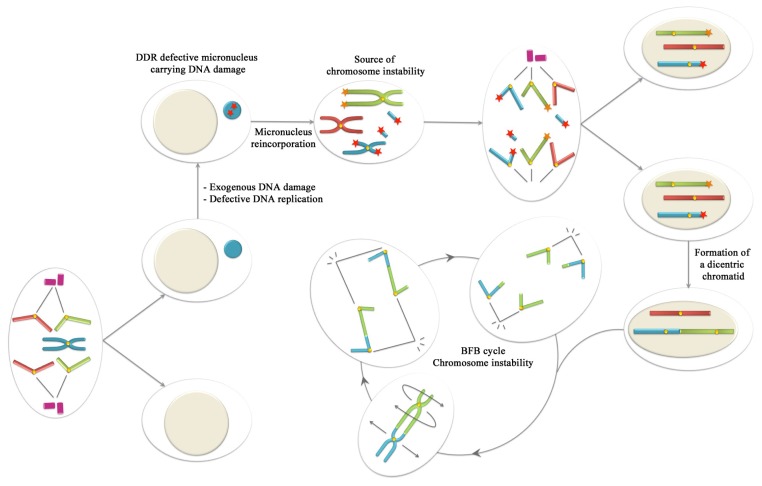
Once a whole-chromosome containing micronucleus is formed, it may accumulate significant DNA damage due to exposure to exogenous damage or due to defective DNA replication. As DDR is defective in micronuclei, the damage will remain unrepaired. As recently demonstrated, micronuclei can reincorporate to the main nucleus after nuclear envelope breakdown. Subsequently, the damaged chromosome may be a source for chromosome reorganizations under certain circumstances. For instance, if chromosomes with dysfunctional telomeres are present, during the next G1-phase, the non-homologous end joining (NHEJ) can join the micronucleus-derived damaged-chromatid with the one lacking telomeres leading to the formation of a dicentric chromatid. After DNA replication and once in mitosis, the dicentric chromosome may initiate breakage-fusion-bridge (BFB) cycles. Therefore, in this situation, micronuclei could be a source for chromosome instability as BFB cycles already are.

The collected data suggest that the DDR machinery is not ready for action anywhere. Only a cell nucleus with an intact envelope structure provides the adequate concentration of proteins for interacting with damaged DNA and triggering an efficient DDR. In the case of micronuclear DNA lesions, the chromatin encapsulated in micronuclei does not benefit from the intricate and efficient web of DDR players of the cell, and chromosome instability would be favored under these circumstances if micronuclei were reincorporated into daughter nuclei. In this sense, it has recently been observed that after nuclear breakdown, some whole-chromosome containing micronuclei might join the other mitotic chromosomes and be distributed to daughter nuclei [32,58]. By photoactivation of the micronuclear chromatin and its subsequent tracking, these authors observed that almost 1/3 of micronuclei reincorporate into daughter nuclei. The remaining micronuclei persisted in cells well into the second generation. Therefore, if lesions are produced in the micronuclear DNA previous to its reincorporation, as DDR is impeded in the micronuclear environment, the damaged micronuclear chromatin could be a source for chromosome instability under certain circumstances (Figure 3). If micronuclei with damaged DNA can finally be reincorporated in the main pool of chromosomes, the presence of unrepaired lesions could certainly increase genomic instability, in terms of gene loss and mutation rate, in the recipient cell. Micronuclei, which have mainly been considered as indicators of ongoing chromosome instability, now emerge as a source of instability at the same time. Altogether, this reveals a new dimension in the significance of micronucleation in the carcinogenesis process.

## Figures and Tables

**Table 1:** Summary of DNA damage response (DDR) factors detected inside micronuclei by different authors (“-” stands for not analyzed, “✘” stands for not found, “✓” stands for found).

DDR factor	Medvedeva *et al*. 2009 [33]	Yoshikawa *et al*. 2009 [31]	Terradas *et al*. 2009 [28]	Crasta *et al.* 2012 [32]
ATM	✓	5%–16%	-	-
DNA-PKcs	-	<1%–7%	-	-
53BP1	✘	5%–6%	14.1%	✓
MRE11	✘	-	27.6%	-
MDC1	✓	-	-	-
RAD17	✘	-	-	-
RAD50	✘	-	-	-
ATR	-	-	-	✓
